# Burnout among Academic Clinicians as It Correlates with Workload and Demographic Variables

**DOI:** 10.3390/bs10060094

**Published:** 2020-05-27

**Authors:** Aussama K. Nassar, Susan Reid, Kamyar Kahnamoui, Faiz Tuma, Abdul Waheed, Meghan McConnell

**Affiliations:** 1 Department of Surgery, Stanford University, Stanford, CA 94305, USA; 2 Department of Surgery, McMaster University, Hamilton, ON L8S4L8, Canada; reid@mcmaster.ca (S.R.); kahnam@mcmaster.ca (K.K.); 3 Central Michigan, University College of Medicine, Mt Pleasant, MI 4885, USA; 4 Brandon Regional Hospital, Brandon, FL 33511, USA; namawaheed@gmail.com; 5 Department of Innovation in Medical Education, University of Ottawa, Ottawa, ON K1N6N5, Canada; mcconn@mcmaster.ca; 6 Department of Anesthesiology and Pain Medicine, University of Ottawa, Ontario, ON K1N6N5, Canada

**Keywords:** burnout, clinicians, factor analysis, Maslach inventory, regression analysis

## Abstract

Burnout syndrome (BOS) in academic physicians is a psychological state resulting from prolonged exposure to job stressors. It leads to a decline in overall job performance, which could result in misjudgment and serious clinical errors. The current study identifies the prevalence, as well as the potential demographic and workload variables that contribute significantly to BOS in academic clinicians. We distributed a modified version of the Maslach Burnout Inventory (MBI) scale to the academic clinicians in our institution; 326/900 responded, with 56.21% male and 43.46% female. The MBI scale comprised of three dimensions of burnout: emotional exhaustion (EE), depersonalization (DP), and personal accomplishment (PA). Higher scores in EE and DP and lower scores in PA were associated with a higher risk for burnout. In considering the work-life of academic clinicians, this study used a modified version of the MBI to reflect three hypothesized sources of burnout: interactions with students/trainees, interactions with patients, and interactions with administration, as reflected in these three dimensions. Along both the EE and DP dimensions of the MBI, burnout was highest for interactions with administration (51% and 44.8%), moderate for interactions with patients (26.4% and 34.5%), and lowest for interactions with students (11.7% and 9.8%). The highest scores along the personal accomplishment component was found for interactions with students and patients (33.7% and 33.4%). Regression analyses identified several factors associated with higher scores on the EE and DP scales: younger age, surgical specialty, low academic rank, academic main practice, female gender, numerous night shifts, and living alone. Furthermore, higher patient volume contributed significantly to the increasing PA. This study suggests that administrative interaction contributes significantly to burnout amongst physicians, followed by patient care and trainees. Furthermore, surgeons, females, single, early career, and younger faculty staff members are at higher risk of suffering from burnout. Further studies are needed to characterize the nature of administrative interactions that contribute to burnout and to solidify other contributing variables.

## 1. Introduction

Burnout was originally defined as “the extinction of motivation or incentive, especially where one’s devotion to a cause or relationship fails to produce the desired results” [1,2]. The term burnout was first used by Californian poverty lawyers to describe peers who exhibited signs of gradual cynicism and emotional exhaustion [3]. Around the same time, Maslach, a social psychologist, focused on stressors among workers in human services and how they coped with such stressors [3,4]. Later on, Maslach and colleagues conceptualized burnout along three dimensions: emotional exhaustion, depersonalization, and personal accomplishment [4].

Employment is generally perceived as an essential element of the overall well-being of an individual. In developed countries, burnout syndrome (BOS) is ranked next to cardiovascular disease and diabetes in prevalence [5,6]. America’s Physicians Practice Patterns Perspectives reported that 78% of physicians had BOS in 2018. This was a 4% increase since 2016. The British Medical Association survey in 2019 demonstrated that 80% of doctors were suffering from BOS [7]. Moreover, BOS often begins when workers have unrealistic expectations of themselves, others, and their career development [8,9,10]. The failure to meet these expectations leads to maladaptive coping strategies, which subsequently results in chronic job stress and, eventually, burnout [8,9,11]. Although it is challenging to classify the symptoms of BOS precisely, the BOS symptoms can be categorized into physical, affective, cognitive, behavioral, and motivational components [12].

Burnout is commonly observed in individuals who work with people, particularly in educators at all levels and healthcare workers, whose careers are characterized by high levels of work-related stress [11]. Burnout is a global concern with deleterious effects on an individual’s psychological and physical health and on an organization as a whole [13]. Understanding the factors that contribute to burnout is essential for creating a healthy and productive workplace.

Clinician faculty educators are at significant risk of burnout, since many are practicing clinicians and researchers expected to handle the pressures associated with multiple roles simultaneously [14].

Educators have been particularly prone to burnout and well-studied in a variety of educators, including elementary [15,16], middle school [17,18], high school [15], and university teachers [18,19]. The prevalence of burnout amongst physicians has been extensively researched [20,21]. When physicians take the educator role (i.e., academic physicians), this might put them at a higher risk of burnout [22]. Administrative duties—including the Electronic Health Record (EHR)—with insufficient documentation time have been linked to BOS in academic clinicians [23]. In a study done on academic physicians, only 3% of the physicians reported administration work to be meaningful [20]. Thus, the present study hypothesized three main sources of burnout among academic clinicians, which are: interactions with patients, trainees, and/or administration.

The current study aims to measure the prevalence of burnout within the Faculty of Health Sciences at McMaster University—a tertiary academic referral health center in Ontario, Canada. The study also aims to investigate potential demographic and workload variables that contribute to self-reported measures of burnout.

We hypothesize that administrative interaction serves as a main source of burnout compared to interaction with trainees and patients.

## 2. Methods

A Research Ethics Board (REB) approval (13-800) was obtained prior to the study commencement for the study protocol, survey, recruitment email, and poster. The participants’ personal information was not reported in any of the findings. Data for this study were collected from February to July, 2014.

### 2.1. Study Population

The survey was completed by 326 clinical academic faculty members in clinical specialties working at McMaster University in the departments of Medicine, Critical Care, Surgery, Family Medicine, Obstetrics, and Gynecology, Pediatrics, Anesthesia, Psychiatry, Emergency Medical Oncology, Radiology, and Pathology (Table 1). The total population surveyed was approximately 900, while our sample that responded was 326. The survey response rate was 36.2%. The highest response rate was in Surgery (60%) and lowest in Medicine (38%).

The specialties were further polarized into two main categories: surgically focused specialties (Surgery, Obstetrics, and Gynecology and Anesthesia), which represented 35%, and non-surgically focused specialties (Medicine, Critical Care, Family Medicine, Pediatrics, Psychiatry, Emergency Medicine, Pathology, Radiology, and Medical/Radiation Oncology), representing 65%. The study population was further categorized based on their academic rank, their highest level of education, living status, gender, and marital status (Table 1).

### 2.2. Instrument Used for the Survey

**Figure 1:**
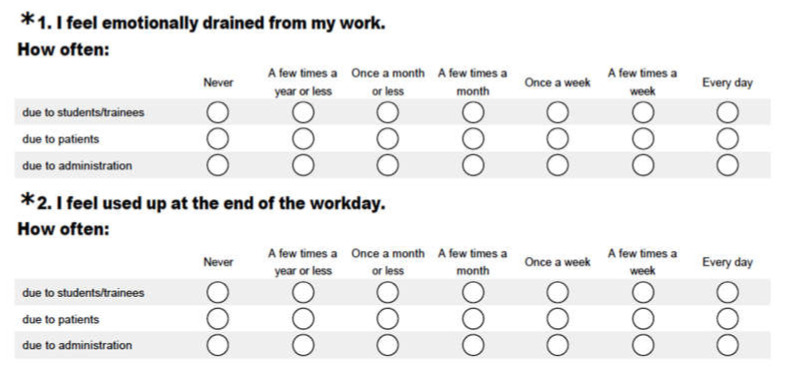
A section of the modified MBI scale (with permission).

The Maslach Burnout Inventory (MBI) was used for measuring burnout among academic healthcare providers (Table 2). The original version of MBI consisted of 22 items, each composed of a seven-point rating scale indicating the frequency of such an experience. To fit the goal of the study, an innovative MBI scale modification was performed with permission from the publisher (Mind Garden^®^). The modification of the scale included the three hypothesized categories of burnout—trainees, patients, and administration—within the same modified version on the same scale administered at the same setting (Figure 1).

### 2.3. Data Collection

Survey Monkey^®^, a web-based survey platform, was used for data collection. The survey link was distributed to participants via email by their corresponding department chairs.

#### Statistical Analysis

A statistical analysis was performed using SPSS Statistical Software 22, (IBM, Chicago, Illinois, USA). All statistical tests were two-tailed, and *p*-values < 0.05 were considered statistically significant. An exploratory factor analysis was used to investigate the construct validity for the modified scale of the three sources of interactions (trainees, patients, or administration) to each of the 22 items of the scale. A multiple regression analysis was carried out to examine the contribution of each of the study variables and how these variables explained the variance of the burnout scores.

These variables included age, gender, marital status, living situation, total years of work experience as faculty, majority of practice community vs. academic, academic rank, specialty, level of higher education achieved, number of night/shifts call per month, average number of patients, patient load per month, number of active research projects the physicians were currently involved in, nature of work, and whether the job was full-time or part-time.

## 3. Results

### 3.1. Psychometric Properties of MBI: Psychometric Properties of the Scale Were Tested to Ensure That Our Modified Scale Bore the Same Dimensionality as That of the Original Unmodified MBI Scale

#### 3.1.1. Construct Validity Index

A principal axis factoring with Varimax rotation was used to investigate the dimensionality of MBI according to the population of the study and to give an index of construct validity (see Appendix A Table A1, Table A2 and Table A3). The factor retention criteria were (1) Eigenvalues greater than 1, (2) evidence from the screen plots, and (3) factor loadings greater than 0.30. A separate factor analysis was conducted for all three categories of responses: due to trainees, due to patients, and due to administration. The three prerequisites for principal axis factoring that the study met were:

1. The determinant analysis was larger than zero for all responses, which were deemed significant.
2. Kaiser–Meyer–Olkin (KMO) of sampling adequacy: KMO for the category due to students was 0.92, due to patients was 0.92, and due to administration was 0.91.
3. Bartlett’s test for sphericity was significant for the three responses (*p* < 0.001).

The extracted sum squared loading indicated what percentage of the variance in an original variable was explained by a factor (Table 3).

#### 3.1.2. Reliability (Internal Consistency)

Nine internal consistencies (Cronbach’s alpha) indexes were calculated and are presented in Table 4. The results indicated that the reliability of the modified scale ranged between 0.86–0.97 (good–excellent) across the three types of human interactions.

#### 3.1.3. Burnout Prevalence

To estimate the prevalence of burnout among the academic clinicians, the burnout percentages for the three types of interaction categories (interactions with trainees, with patients, and with administration) were calculated and are presented in Table 5. Academic clinicians reported higher levels of burnout due to interactions with administration relative to that caused by patients and trainees in both emotional exhaustion (EE), depersonalization (DP), and personal accomplishment (PA) (percentages are interpreted in reverse to the other two subscales). The mean squares with standard deviation for the three types of interaction categories were calculated and are presented in Table 6.

### 3.2. Variables Contributing to Burnout

A regression analysis was used to determine the contributing variables for burnout syndrome. Thirteen variables were included as predictors for burnout: age, gender, marital status, living situation, years of work experience, practice type (community vs. academic), academic rank, specialty, education achieved, number of work hours/month, patient load per month, number of active research projects, and full or part-time practice.

To satisfy the requirement of multiple regressions, categorical and ordinal data variables were converted to dummy variables of two values: 0 and 1. The sample size variable ratio was calculated to be 25, which is acceptable for a regression analysis.

The following variables were converted to dummy variables:

1. Marital status: single and separated were coded as 0 and married as 1.
2. Specialty: surgical specialties (Surgery, OBGYN, and Anesthesia) were coded as 0, while non-surgical specialties (Medicine, Critical Care, Family Medicine, Pediatrics, Psychiatry, Emergency Medicine, and Medical Oncology) were coded as 1.
3. Academic rank: professor and associate professor were coded as 0; while assistant professor, associate clinical professor, assistant clinical professor, and clinical scholar were coded as 1.
4. Living situation: alone was 0, while living with others was 1.
5. Educational level: postgraduate education, Ph.D., MBA, Masters, and diploma was coded as 0, while no postgraduate degree was coded as 1.

Multiple regressions with stepwise methods in entering the predictor (independent) variable were used. The results are summarized in Table A4, Table A5 and Table A6 (see Appendix A).

#### 3.2.1. Emotional Exhaustion Dimension of Burnout

Multiple regression was used to compare three sources of EE (burnout due to trainees, burnout due to patients, and burnout due to administration) according to the contribution of the 13 predictors, and to explain the variance of the EE burnout.

The results of the multiple regression were as follows (Table A4 in Appendix A).

Specialty and age contributed to the variance of the emotional exhaustion subscale of burnout due to interaction with trainees. These two variables explained 6% of the EE burnout variance. Four variables (age, number of night/shifts per month, living alone or with others, and specialty) accounted for 10% of the EE due to patients’ variance. Five variables (specialty, number of night/shifts per month, the setting of practice community versus hospital, marital status, and academic rank) contributed significantly to the regression model and accounted for 20% of the variance of EE due to interactions with administration.

Specialty contributed to the variance of all the three types of interactions of the EE burnout subscale. The beta weights of this variable in EE burnout due to patients’ interaction (−0.12) were lower than both EE burnout due to administration interactions (−0.22) and EE burnout due to trainees’ interaction (−0.22). The negative direction of beta indicated that non-surgical specialties negatively contributed to the EE subscale of burnout compared to surgical specialties.

#### 3.2.2. Depersonalization Dimension of Burnout

To analyze the DP burnout dimension, the same 13 variables were used as predictors. The same scales of measurements and coding of the dummy variables were used. Multiple regressions with stepwise methods in entering the predictors’ variables were used to analyze DP burnout. The results for the DP burnout are summarized in Table A5 in the Appendix A).

Three out of the 13 variables contributed to the variance of the students’ DP burnout. The three variables were: specialty, number of night shifts per month, and setting of practice (community versus academic). The contribution of these three variables was 0.14% of the DP burnout variance. Five variables (specialty, age, academic degree, marital status, and night shifts) explained 0.15% of the variance of DP due to patients. Six variables (specialty, night shifts, setting of practice, academic position, marital status, and gender) contributed significantly to DP and explained 0.25% of the variance of DP due to administration.

Only specialty and number of night shifts contributed to the variance of all three categories of the sources of DP burnout. The beta weights of these variables were higher in DP burnout due to administration in both categories (specialty: −0.25 and night shift: 0.22) than both DP burnout due to students (specialty: −0.23 and night shifts: 0.19) and due to patients (specialty: −0.20 and night shifts: 0.13). The results indicated a negative direction of beta for specialty and a positive direction for night shifts in all the three categories of DP. Furthermore, DP burnout for the non-surgical specialty was less than the surgical specialty. This suggests that the more night shifts, the higher the DP burnout for the three categories.

#### 3.2.3. Personal Accomplishment Dimension of Burnout

To analyze the PA burnout dimension, the same 13 variables were used as predictors (Table A6 in Appendix A). The results of PA burnout were as follows:

Two variables (specialty and number of in-patients per month) out of the 13 predictors contributed to the students’ PA burnout variance. These variables explained 5% of the PA burnout variance. Specialty, setting of practice (community versus academic), number of in-patients per month, and academic rank explained 14% of the PA due to patients. Specialty and setting of practice (community versus academic) contributed significantly to PA and explained 0.12% of the variance of PA due to administration.

Only specialty contributed to the variance of all three categories of the interactions of PA burnout. The beta weights of this variable were higher in PA burnout due to administration (0.30) than both due to students (0.16) and due to patients (0.19). The results indicated a significant positive direction of beta for specialty in all three main burnout interactions of PA burnout. This suggests that PA for the non-surgical specialties is higher than the surgical specialties for the three categories of burnout.

## 4. Discussion

Academic clinicians have more human interactions in comparison with non-academic clinicians: specifically, interactions with trainees and administration. Therefore, academic clinicians were hypothesized to be at a higher risk of burnout. We hypothesized that the three main sources of burnout in academic physicians are: interactions with trainees, with patients, and with administration. This study utilized a novel modification of Maslach’s Burnout Inventory to identify the main sources and prevalence of burnout for academic clinicians. In addition, we investigated the contributing factors to burnout, as it relates to demographic and workload variables.

Due to this novel modification, the psychometric properties of the scale were tested to ensure that our modified scale bore the same dimensionality as that of the original unmodified MBI scale.

### 4.1. Burnout Source and Prevalence in Academic Clinicians

This is the first study investigating the three main interactions related to burnout in a single scale in one administration. Our study showed that the least source of burnout in academic clinicians was associated with trainee interactions, followed by patient interactions, while the highest burnout levels were related to interactions with administration. The impact of administrative interactions on burnout symptoms was a particularly interesting and novel finding. Respondents may have perceived interactions with administration in different ways: e.g., office work, EHR (Electronic Health Records), secretaries, charts, academic, or hospital roles. Further studies are required to pinpoint the specific administrative interactions related to burnout in academic clinicians.

A study on 465 academic clinicians showed that the majority (68%) reported patient care as the aspect of work they found most meaningful, followed by research (19%), and education (9%). The least was administration (3%). Those spending less than 20% of their time on the most meaningful activity had higher rates of burnout on the multivariate analysis. This study concluded that spending more time on administrative responsibilities predisposes academic physicians to burnout. EHR were shown to be a source of burnout [24]. Although patient care was shown to be most meaningful to academic physicians [23], it was also a significant source of burnout, depending on the nature of specialty and workload.

Trainee interactions demonstrated the least source of burnout for academic clinicians in the present study. This might be because trainees (medical students, residents, and fellows) share a great deal of the workload in an academic institute. In addition, trainees work under the supervision of the academic physician; therefore, they are under their authority, which may render the interaction less stressful for clinicians.

### 4.2. Variables Contributing to Burnout

One of the main objectives of this study was to investigate the independent variables contributing to burnout to implement better interventional methods to target these variables in the workplace.

Our results showed that younger physicians reported higher levels of emotional exhaustion due to interactions with trainees and patients, as well as more depersonalization in response to patient interaction. This is consistent with previous research showing that burnout is more prevalent in younger workers [25,26] and younger physicians [26,27,28]. A possible explanation is that younger workers are less experienced; they may become more cognitively overwhelmed with the workload of the “routine workday” of a senior physician. Moreover, for technical specialties, such as surgery, young surgeons may still be on the steep portion of their technical learning curve, with associated performance anxiety and fear of complications.

The current study revealed that females rated higher on the DP subscale due to interactions with administration than males. The Physicians Work Life study of a random stratified sample of 6000 physicians revealed that female physicians were 60% more likely to report burnout than male physicians [29]. Furthermore, female physicians generally have more domestic responsibilities than male physicians, which might contribute to burnout [30,31].

In this study, marital status was a contributing variable to burnout, with single physicians being more prone to burnout. It was hypothesized that the reason married workers experienced lower levels of burnout is that they were usually older, psychologically mature, and stable. Furthermore, they had higher interpersonal skills and were more skillful in problem-solving and adaptability due to their involvement with their families [11].

Certain specialties are more prone to burnout. This study showed that surgical specialties contributed to burnout. Studies from various surgical subspecialty societies in the US estimated that burnout rates among surgeons ranged from 30–48% [32,33]. Surgical specialties have been reported to be stressful [34,35,36,37]. Personality characteristics may be related to the higher levels of burnout associated with surgical specialties. For example, although some surgeons may believe that they are more resilient than their colleagues, the inherent personality traits that define a surgeon put them at an increased risk of distress and burnout [26,33]. Since research has shown that the number of medical errors reported by surgeons is related to the level of burnout and the quality of their mental life [38], it is imperative to continue to study burnout to better understand its origins and prevention.

Lower academic rank was positively correlated with burnout and inversely correlated with PA due to patient interactions. This may be due to a similar explanation as the age above.

Patient load was reported to play a critical role in burnout amongst clinicians. In this study, the number of patients under the care of an academic clinician per month had a significant positive correlation with PA due to the student and patient interactions. This corroborates other studies showing that the most meaningful aspect of an academic clinician’s job is patient care [38]. Patient care gives clinicians a sense of personal accomplishment, since it is the essence of being a doctor. In academic settings, an increased patient load may be associated with increased trainee assistance in sharing the workload and, hence, more opportunities for teaching interactions. This could lead to an increased sense of personal accomplishment.

## 5. Limitations

This study was a single center study, and, therefore, the results could be institution-specific. Another limitation was that the exact survey response rate for this study could not be accurately calculated. This was because the email distribution list was outdated: it included faculty that had vacated their position, retired, or ones with duplicated emails, including that of their admins. Furthermore, some were non-clinical faculty (PhD, researchers, etc.). This led to an estimated response rate of 36.2%.

## 6. Conclusions

This study investigated the prevalence of burnout and the potential etiological variables of the work-related stressors of burnout in academic physicians in a single academic center. Our results demonstrated, as hypothesized, that academic clinicians have an increased risk of burnout syndrome, and this is primarily due to interaction with administration.

This study used a novel modification of Maslach’s Burnout Inventory to identify the potential etiological factors associated with burnout in clinician educators. Three different interactions were queried: students/trainees, patients, and administration. Our results indicated that the interactions with the administration were the primary source of burnout for faculty members, followed by patients, and, lastly, trainees. Our results also demonstrated that burnout is more common in young physicians, surgical specialties, and among individuals who are single or separated. More studies in this area are required to solidify the contributing variables of burnout in our healthcare system.

In addition to having psychological implications, burnout syndrome causes workers to become disengaged in their work and become less confident. More seriously, burnout has been linked to significant clinical errors. This has substantial repercussions on patient care, as well as the quality of training for future physicians. Treating burnout is resource-intensive and still experimental. The best remedy is prevention. Therefore, it is essential to investigate the contributing variables to burnout for academic clinicians and to develop effective preventative measures to render the work environment less stressful, more productive, and, ultimately, transpire to better patient care.

## Figures and Tables

**Table 1:** Study Demographics (*n* = 326).

Gender	N (%)
Male	183 (56.21)
Female	142 (43.46)
**Marital Status**	
Single	37 (11.37)
Married	263 (80.60)
Separated/divorced	26 (8.03)
**Base Specialty**	
Medicine	101 (31.15)
Critical Care	10 (2.95)
Surgery	66 (20.33)
Family Medicine	12 (3.61)
OBGYN	14 (4.26)
Pediatrics	15 (6.56)
Anesthesia	30 (9.18)
Psychiatry	26 (7.87)
Emergency Medicine	17 (5.25)
Medical/radiation Oncology	11 (3.28)
Radiology	5 (1.64)
Pathology	13 (3.93)
**Academic Appointment**	
Professor Emeritus	3 (0.99)
Professor	35 (10.60)
Associate Professor	97 (29.80)
Assistant Professor	77 (23.51)
Associate Clinical Professor	40 (12.25)
Assistant Clinical Professor	66 (20.20)
Clinical Scholar	9 (2.65)
Locum	0 (0.00)
**Living Situation**	
Alone	37 (11.30)
With Partner	90 (27.57)
With family	199 (61.13)
Shared Accommodation	0 (0.00)
Hotel or Guesthouse	0 (0.00)
**Highest Level of Education**	
Ph.D.	36 (11.11)
MBA	7 (2.29)
M.Sc.	76 (23.20)
Diploma	19 (5.88)
M.D. (only)	150 (46.08)

**Table 2:** Maslach Burnout Inventory (MBI): Major Instrument Used in Measuring Burnout among Healthcare Workers.

Name	Maslach Burnout Inventory (MBI)
**Content**	22 items
	7 response categories
**Domains**	Emotional Exhaustion (EE)
	Depersonalization (DP)
	Personal Accomplishment (PA)

**Table 3:** Extracted Sum Squared Loading for the Modified MBI scale.

Burnout Source Subscale	Administration	Patients	Students/Trainees
**EE**	53.48%	36.04%	36.56%
**DP**	12.12%	18.34%	14.81%
**PA**	3.56%	5.07%	5.41%

**Table 4:** Internal Consistency (Cronbach’s alpha Values).

Burnout Source Subscale	Administration	Patients	Students/Trainees
**EE**	0.97	0.93	0.93
**DP**	0.92	0.86	0.82
**PA**	0.90	0.89	0.88

**Table 5:** Burnout prevalence among the three major sources of human interactions. Percentages indicate the levels of burnout, as defined by Maslach and Jackson (1986).

Burnout Source Subscale	Administration	Patients	Students/Trainees
**EE**	51.8%	26.4%	11.7%
**DP**	44.8%	24.5%	9.8%
PA *	16.3%	33.4%	33.7%

* Percentages are interpreted in reverse to the EE and DP scales.

**Table 6:** Means and standard deviations of the burnout scores for the three types of interactions.

Burnout Source Subscale	Administration	Patients	Students/Trainees
**EE**	27.31 (15.93)	19.46 (11.31)	13.3 (10.44)
**DP**	12.52 (9.16)	7.59 (6.00)	5.34 (4.95)
PA *	18.96 (11.57)	35.25 (9.48)	34.42 (9.58)

Standard deviations appear in parentheses below means.* PA values interpreted in reverse to the EE and DP scales.

## Appendix A

**Table A1:** Rotated factor Matrix for Burnout due to Student’s Interaction.

Due to Students/Trainees	Factor		
	EE	PA	DP
I feel emotionally drained from my work (1)	0.873	−0.093	0.036
I feel used up at the end of the workday (2)	0.877	−0.066	0.158
I feel fatigued when I get up in the morning and have to face another day on the job (3)	0.773	−0.094	0.26
Working with people all day is really a strain for me (6)	0.678	−0.174	0.366
I feel burned out from my work (8)	0.775	−0.068	0.336
I feel frustrated by my job (13)	0.757	−0.103	0.208
I feel I’m working too hard on my job (14)	0.66	0.104	0.23
I feel like I’m at the end of my rope (20)	0.569	−0.18	0.49
I feel very energetic (12)	−0.371	0.722	−0.047
I can easily create a relaxed atmosphere with my students (17)	−0.184	0.712	−0.182
I feel exhilarated after working closely with my students (18)	−0.224	0.714	0.156
I have accomplished many worthwhile things in this job (19)	−0.064	0.774	−0.057
I don’t really care what happens to some of my students (15)	0.148	−0.273	0.71
I feel students blame me for some of their problems (22)	0.299	−0.06	0.473
I’ve become more callous (emotionally hardened) toward people since I took this job (10)	0.471	−0.18	0.555
I worry that this job is hardening me emotionally (11)	0.512	−0.064	0.591
Working with people directly puts too much stress on me (16)	0.481	−0.18	0.481

Extraction Method: Principal Axis Factoring.

**Table A2:** Rotated Factor Matrix for Burnout due to Patient’s.

Due to Patients	Factor		
	EE	PA	DP
I feel emotionally drained from my work (1)	0.853	0.006	0.163
I feel used up at the end of the workday (2)	0.832	0.053	0.194
I feel fatigued when I get up in the morning and have to face another day on the job (3)	0.775	−0.062	0.32
Working with people all day is really a strain for me (6)	0.701	−0.139	0.33
I feel burned out from my work (8)	0.797	−0.082	0.351
I feel frustrated by my job (13)	0.722	0.063	0.334
I feel I’m working too hard on my job (14)	0.642	0.173	0.147
I can easily understand how my patients feel about things (4)	0.149	0.564	−0.23
I deal very effectively with the problems of my patients (7)	0.13	0.757	−0.159
I feel I’m positively influencing other people’s lives through my work (9)	0.023	0.748	−0.09
I feel very energetic (12)	−0.366	0.688	−0.003
I can easily create a relaxed atmosphere with my patients (17)	0.006	0.761	−0.34
I feel exhilarated after working closely with my patients (18)	−0.175	0.644	0.155
I have accomplished many worthwhile things in this job (19)	−0.042	0.735	−0.08
In my work, I deal with emotional problems very calmly (21)	0.084	0.709	−0.301
I feel I treat some patients as if they were impersonal objects (5)	0.282	−0.192	0.653
I’ve become more callous (emotionally hardened) toward people since I took this job (10)	0.447	−0.054	0.621
I worry that this job is hardening me emotionally (11)	0.458	−0.179	0.672
I don’t really care what happens to some of my patients (15)	0.198	−0.277	0.645
I feel patients blame me for some of their problems (22)	0.258	−0.046	0.55
Working with people directly puts too much stress on me (16)	0.465	−0.177	0.512
I feel like I’m at the end of my rope (20)	0.507	−0.216	0.604

Extraction Method: Principal Axis Factoring.

**Table A3:** Rotated Factor Matrix for Burnout due to Administration.

Due to Administration	Factor		
	EE	PA	DP
I feel emotionally drained from my work (1)	0.861	−0.222	0.028
I feel used up at the end of the workday (2)	0.883	−0.184	0.112
I feel fatigued when I get up in the morning and have to face another day on the job (3)	0.873	−0.207	0.119
I feel I treat some administration as if they were impersonal objects (5)	0.512	−0.18	0.546
Working with people all day is really a strain for me (6)	0.783	−0.256	0.28
I feel burned out from my work (8)	0.887	−0.23	0.063
I’ve become more callous (emotionally hardened) toward people since I took this job (10)	0.747	−0.207	0.35
I worry that this job is hardening me emotionally (11)	0.774	−0.206	0.325
I feel frustrated by my job (13)	0.847	−0.239	0.053
I feel I’m working too hard on my job (14)	0.831	−0.04	0.104
Working with people directly puts too much stress on me (16)	0.685	−0.319	0.36
I feel like I’m at the end of my rope (20)	0.794	−0.286	0.315
I feel administration blame me for some of their problems (22)	0.645	−0.164	0.465
I can easily understand how my administration feel about things (4)	−0.106	0.502	−0.438
I deal very effectively with the problems of my administration (7)	−0.13	0.64	−0.422
I feel I’m positively influencing other people’s lives through my work (9)	−0.187	0.817	−0.116
I feel very energetic (12)	−0.29	0.727	−0.049
I can easily create a relaxed atmosphere with my administration (17)	−0.277	0.644	−0.378
I feel exhilarated after working closely with my administration (18)	−0.148	0.74	−0.016
I have accomplished many worthwhile things in this job (19)	−0.237	0.801	−0.162
I don’t really care what happens to some of my administration (15)	0.56	−0.308	0.557
In my work, I deal with emotional problems very calmly (21)	−0.11	0.463	−0.478

Extraction Method: Principal Axis Factoring.

**Table A4:** Linear Regression for emotional exhaustion (EE) subscale.

**EE Students**	**Model 1**			**Model 2**											
**Variable**	**B**	**SE B**	***β***	**B**	**SE B**	***β***									
Constant	16.64	1.046		23.05	3.264										
Specialty	−4.75	1.31	−0.22	−4.68	1.30	−0.22									
Age				−0.14	0.07	−0.12									
R^2^		0.05			0.06										
F		13.161 **			8.808 **										
**EE Patients**	**Model 1**			**Model 2**			**Model 3**			**Model 4**					
**Variable**	**B**	**SE B**	***β***	**B**	**SE B**	***β***	**B**	**SE B**	***B***	**B**	**SE B**	***β***			
Constant	30.973	3.348		27.428	3.571		36.376	5.156		38.431	5.219				
Age	−0.221	0.069	−0.195	−0.189	0.069	−0.166	−0.181	0.069	−0.159	−0.180	0.068	−0.158			
Night shift				0.341	0.129	0.161	0.337	0.128	0.159	0.305	0.128	0.144			
Living condition							−4.926	2.064	−0.142	−4.990	2.052	−0.144			
Specialty										−2.779	1.364	−0.123			
R^2^		0.04			0.06			0.08			0.10				
F		10.271 **			8.75 **			7.841 **			7.020 **				
**EE Administration**	**Model 1**			**Model 2**			**Model 3**			**Model 4**			**Model 5**		
**Variable**	**B**	**SE B**	***β***	**B**	**SE B**	***β***	**B**	**SE B**	***B***	**B**	**SE B**	***β***	**B**	**SE B**	***β***
Constant	33.705	1.56		28.453	1.921		35.473	3.368		44.422	5.153		38.182	5.958	
Specialty	−9.491	1.951	−2.88	−8.460	1.9	−0.257	−7.670	1.907	−0.233	−7.618	1.892	−0.231	−7.351	1.885	−0.223
Night Shifts				0.785	0.178	0.255	0.68	0.181	221	0.662	0.18	0.215	0.649	0.179	0.211
Community vs. academic							−5.612	2.222	−0.150	−5.255	2.21	−0.140	−5.564	2.201	−0.148
Marital status										−5.157	2.261	−0.129	−4.845	2.252	−1.21
Academic position													3.724	1.817	0.115
R^2^		0.08			0.15			0.17			0.18			0.197	
F		23.656 **			22.387 **			17.360 **			14.532 *			12.610 **	

* *p* less than 0.05 ** less than 0.01.

**Table A5:** Linear Regression for depersonalization (DP) subscale.

**DP Students**	**Model 1**			**Model 2**			**Model 3**											
**Variable**	**B**	**SE B**	***B***	**B**	**SE B**	***β***	**B**	**SE B**	***β***									
Constant	7.411	0.498		5.926	0.618		8.072	1.085										
Specialty	−2.988	0.623	−0.285	−2.697	0.611	−0.257	−2.455	0.614	−0.234									
Night shift				0.222	0.057	0.226	0.19	0.058	0.193									
Community vs. Academic							−1.715	0.716	−0.144									
R^2^		0.08			0.131			0.140										
F		23.007 **			19.625 **			15.236 **										
**DP Patients**	**Model 1**			**Model 2**			**Model 3**			**Model 4**			**Model 5**					
**Variable**	**B**	**SE B**	***B***	**B**	**SE B**	***β***	**B**	**SE B**	***β***	**B**	**SE B**	***β***	**B**	**SE B**	***β***			
Constant	9.716	0.596		15.508	1.837		12.552	2.158		15.828	2.525		14.363	2.603				
Specialty	−2.829	0.746	−0.228	−2.768	0.732	−0.224	−2.679	0.726	−0.216	−2.629	0.719	−0.212	−2.455	0.719	−0.198			
Age				−0.123	0.037	−0.197	−0.122	0.036	−0.196	−0.110	0.036	−0.177	−0.097	0.037	−0.156			
Academic Degree							1.802	0.708	0.149	1.808	0.702	0.15	1.649	0.701	0.136			
Marital										−2.145	0.88	−0.143	−2.070	0.875	−0.138			
Night shifts													14.363	2.603	0.125			
R^2^		0.052			0.091			0.113			0.133			0.148				
F		14.370 **			12.995 **			11.002 **			9.892 **			8.907 **				
**DP administration**	**Model 1**			**Model 2**			**Model 3**			**Model 4**			**Model 5**			**Model 6**		
**Variable**	**B**	**SE B**	***B***	**B**	**SE B**	***β***	**B**	**SE B**	***β***	**B**	**SE B**	***β***	**B**	**SE B**	***β***	**B**	**SE B**	***β***
Constant	16.936	0.901		13.435	1.1		18.289	1.9		14.002	2.447		18.851	3.346		22.092	3.685	
Specialty	−6.466	1.125	−0.336	−5.749	1.083	−0.299	−5.162	1.082	−0.268	−4.930	1.072	−0.256	−4.910	1.065	−0.255	−4.709	1.063	−0.245
Night Shifts				0.517	0.101	0.287	0.447	0.102	0.248	0.438	0.101	0.243	0.429	0.1	0.238	0.402	0.101	0.223
Community vs. academic							−3.921	1.262	−0.178	−4.172	1.25	−0.190	−3.981	1.245	−0.181	−3.783	1.241	−0.172
Academic position										2.807	1.029	0.149	2.665	1.024	0.141	2.927	1.026	0.155
Marital status													−2.668	1.264	−0.114	−3.131	1.277	−0.134
Gender																−2.132	1.045	−0.114
R^2^		0.11			0.19			0.22			0.23			0.24			0.25	
F		33.053 **			31.160 **			24.686 **			20.840 *			17.786 **			15.699 **	

* *p* < 0.05 ** *p* < 0.01.

**Table A6:** Linear Regression for personal accomplishment (PA) subscale.

**PA Students**	**Model 1**			**Model 2**								
**Variable**	**B**	**SE B**	***β***	**B**	**SE B**	***B***						
Constant	33.695	0.873		33.096	0.903							
Specialty	3.026	1.092	0.169	2.772	1.088	0.155						
MRP/Month				0.035	0.015	0.142						
R^2^		0.029			0.049							
F		7.672 **			6.637 **							
**PA Patients**	**Model 1**			**Model 2**			**Model 3**			**Model 4**		
**Variable**	**B**	**SE B**	***β***	**B**	**SE B**	***B***	**B**	**SE B**	***β***	**B**	**SE B**	***β***
Constant	33.737	0.824		29.428	1.549		27.975	1.617		31.894	2.233	
Specialty	4.513	1.031	0.262	3.881	1.031	0.225	3.482	1.028	0.202	3.316	1.019	0.192
Community vs. Academic				3.826	1.173	0.195	4.52	1.185	0.23	4.649	1.174	0.237
MRP/Month							0.039	0.014	0.165	0.037	0.014	0.157
Academic position										−2.455	0.975	−0.145
R^2^		0.068			0.098			0.121			0.139	
F		19.176 **			15.263 **			13.003 **			11.537 **	
**PA administration**	**Model 1**			**Model 2**								
**Variable**	**B**	**SE B**	***β***	**B**	**SE B**	***B***						
Constant	14.632	1.133		10.426	2.153							
Specialty	7.892	1.418	0.326	7.275	1.432	0.300						
Community vs. Academic				3.734	1.630	0.135						
R^2^		0.106			0.124							
F		30.983 **			18.368 **							

* *p* < 0.05 ** *p* < 0.01.
